# Antimicrobial resistance knowledge and practices among animal drug vendors in Kano State, Nigeria

**DOI:** 10.3389/fpubh.2026.1793310

**Published:** 2026-04-10

**Authors:** Zainab Bello Dambazau, Hauwa Yusuf Daya, Abiodun Egweanu, Timothy Yapilami Jr, Lorna Williams-Enenche, Ahmed Muhammad Ahmed, Aisha Bello Dambazau, Abdulrahman Muhammad, Arhyel Malgwi

**Affiliations:** 1 Nigeria Field Epidemiology and Laboratory Training Program, FCT, Abuja, Nigeria; 2 One Health Empowerment Initiatives, FCT, Abuja, Nigeria; 3 Nigeria Centre for Disease Control and Prevention, FCT, Abuja, Nigeria; 4 Yobe State Ministry of Agriculture, Damaturu, Nigeria; 5 Charite Universitätsmedizin, Berlin, Germany; 6 Research and Preventive Healthcare Initiative, Abuja, Nigeria; 7 State Ministry of Agriculture, Birnin Kebbi, Kebbi, Nigeria; 8 Capital City University, Kano, Nigeria; 9 Kano State Veterinary Drugs and Agro Allied Association, Kano, Nigeria

**Keywords:** antimicrobial resistance, drug resistance, drug vendors, knowledge, attitudes, practice, Nigeria, One Health, Veterinary Drugs

## Abstract

**Background:**

Antimicrobial resistance (AMR) is a critical One Health challenge, with limited data on the knowledge, attitudes, and practices (KAP) of informal veterinary drug vendors in Nigeria. This study assessed AMR-related knowledge, attitudes, and dispensing practices and their associated factors among registered animal drug vendors in Kano State, Nigeria.

**Methods:**

A cross-sectional survey in December 2024 recruited 133 registered animal drug vendors using a semi-structured questionnaire to collect demographic data and AMR-related knowledge, attitudes, and dispensing practices. One respondent with substantial missing responses was excluded during data cleaning, yielding an analytic sample of 132. KAP levels were classified using a ≥70% cutoff. Data were analyzed using chi-square tests and multivariable logistic regression in R, reporting adjusted odds ratios (AOR) with 95% confidence intervals (CI).

**Results:**

Respondents were predominantly male (99%), urban-based (98.4%), and aged 30–39 years (51.2%), with a mean age of 34.8 years. Adequate knowledge, positive attitudes, and good dispensing practices were reported by 34.8%, 65.2%, and 23.5% of vendors, respectively (*n* = 132). Tertiary education was significantly associated with better knowledge (χ^2^ = 5.36, *p* = 0.0207). Multivariable analysis showed that vendors with up to secondary education had lower odds of adequate knowledge (AOR = 0.43, 95% CI: 0.20–0.94, *p* = 0.0347). Prior AMR training increased the likelihood of adequate knowledge (AOR = 2.21, 95% CI: 1.02–4.82, *p* = 0.0445). Years of experience were not associated with knowledge.

**Conclusion:**

AMR knowledge and, in particular, appropriate dispensing practices remain low among animal drug vendors in Kano State, despite generally positive attitudes. These findings highlight the need for targeted, context-appropriate AMR awareness and stewardship sensitization interventions that strengthen vendor knowledge, reinforce responsible dispensing practices, and support informed decision-making at the point of antimicrobial access, thereby reducing resistance risks at the human–animal interface.

## 1 Introduction

Medicines used for the treatment of diseases are often misused by humans, thereby increasing the present-day crisis of antimicrobial resistance (AMR). This growing crisis has become a major global health threat, responsible for an estimated 1.27 million deaths annually, with nearly 5 million deaths associated with resistant infections worldwide (1). Sub-Saharan Africa bears the highest burden of AMR, driven by a high prevalence of infectious diseases, weak healthcare systems, and limited antimicrobial stewardship programs (2). The region has the world's highest AMR-associated mortality, with 24 deaths per 100,000 people directly attributable to resistance (1). Antibiotic use in hospitals is alarmingly high, ranging from 37.7% in South Africa to 80.1% in Nigeria, with widespread misuse of broad-spectrum antibiotics and poor adherence to treatment guidelines (3).

In Nigeria, AMR is compounded by weak surveillance systems, empirical prescribing, and the emergence of multidrug-resistant bacteria in both clinical settings and food animal production (4, 5). Inappropriate use of antibiotics, antifungals, antivirals, and antiparasitic drugs, often without prescription or for inadequate durations, further accelerates resistance. The misuse of topical agents, including creams and eye drops, also contributes to this problem (5). As resistant pathogens spread easily, they pose a serious threat to the effectiveness of modern medicine.

The burden is particularly evident in low- and middle-income countries (LMICs) where healthcare access is limited and antibiotic use is poorly regulated (6, 7). In Nigeria, over-the-counter availability of antibiotics worsens the situation. National reviews show high rates of multidrug resistance among bacteria from both humans and animals, posing serious threats to treatment effectiveness (4).

However, AMR surveillance in animal health remains fragmented, with limited data from informal veterinary drug markets and livestock production systems (4). Antibiotics are regularly dispensed by both unlicensed and licensed vendors without prescriptions, an illegal but widespread practice (5). In Northern Nigeria, about 40% of licensed community pharmacists dispense antibiotics without prescriptions daily, driven by patient demand and confidence in their knowledge (8).

A systematic review confirmed that patent and proprietary medicine vendors (PPMVs), many poorly monitored, routinely dispense antimicrobials outside regulatory frameworks, exemplifying the failure of existing control mechanisms (9). In Kano State, a key livestock-producing region in northern Nigeria, this risk is heightened (4). Poor access to diagnostics, such as antimicrobial susceptibility testing (AST), forces vendors and farmers to rely on broad-spectrum drugs, increasing selective pressure for resistance (10). Consequently, resistant bacteria circulate between animals, humans, and the environment, amplifying the One Health risk (11).

The problem in Kano is further worsened by weak regulation, economic barriers, and limited stewardship awareness among vendors (9). These practices are driven by profit motives, customer demand, and a lack of enforcement (8). Livestock farmers also misuse antibiotics to prevent disease or promote growth, contributing to residues and resistance in animal products (4). Similar misuse has been reported in Ethiopia, Kenya, and Tanzania, where weak regulations allow over-the-counter antibiotic sales without veterinary oversight (12).

Despite the recognized threat of AMR, there is a critical gap regarding the knowledge and dispensing practices of animal drug vendors in Kano State. Most previous studies in Nigeria have focused on veterinarians, students, or healthcare workers, leaving vendors, both licensed and unlicensed as an under-researched but influential group in AMR emergence (13).

Nigeria has adopted a One Health approach to tackle AMR, yet its effectiveness is hindered at the local level by these systemic weaknesses (14). Encouragingly, studies among veterinary students and practitioners show low baseline awareness but a willingness to learn and improve practices when training is provided (15). Extending such educational interventions to vendors in Kano could significantly reduce inappropriate antimicrobial use.

This study assessed AMR-related knowledge, attitudes, and dispensing practices and their associated factors among registered animal drug vendors in Kano State, Nigeria. The findings will inform targeted interventions and contribute to strengthening One Health antimicrobial stewardship at the animal–human interface.

## 2 Materials and methods

### 2.1 Study design

This was a cross-sectional study using a semi-structured, interviewer-administered questionnaire.

### 2.2 Study area

Kano State, located in northern Nigeria, is the country's second most populous state with an estimated population of 15.46 million (16). The state is a major livestock trade and agricultural hub, with extensive animal markets and veterinary clinics that represent potential hotspots for antimicrobial resistance (AMR) transmission due to intensive antimicrobial use in both human and veterinary sectors (17).

Data were collected in December 2024.

### 2.3 Study population

This study focused on the Veterinary Council of Nigeria (VCN)-registered or Kano State Veterinary Drugs and Agro-allied Association. In this study, “informal” refers to dispensing behaviors and market dynamics (e.g., frequent over-the-counter sales without prescriptions, variable enforcement, and client-driven purchasing), rather than the legal registration status of vendors. Thus, although participants were formally registered, they function within a market context where dispensing practices can be informal.

### 2.4 Sample size and sampling technique

A multistage sampling approach was utilized. In stage one, cluster sampling was used to select a prominent modern veterinary drug market in Kano metropolitan area. This market was chosen because it had a comprehensive list of 236 registered animal drug vendors, maintained by the Veterinary Council of Nigeria (VCN) and the Kano State Veterinary Drugs and Agro-allied Association, which served as the sampling frame.

In stage two, convenience sampling was applied to recruit both wholesalers and retailers from this list, based on their availability and willingness to participate. Using this frame, 133 vendors completed the questionnaire (non-response rate: 43.6%). During data cleaning, one questionnaire with substantial missing responses across the KAP items was excluded, leaving 132 (Table 1) respondents for analysis.

**Table 1:** Sociodemographic characteristics of animal drug vendors in the study (*n* = 132).

Characteristics	Category	Percentage
Age group	20–29	25.2
	30–39	51.1
	40–49	18.3
	>50	5.3
Sex	Male	99.2
	Female	0.8
Location	Urban	99.2
	Rural	0.8
Drug type sold	Animals	98.5
	Animals/human	1.5
Job role	Manager	71.2
	Shopkeeper	27.3
	Internship	1.6
Years in business	1–3 years	15.4
	4–6 years	42.3
	>10 years	42.3
Mean age (± SD): 34.8 (± 2.06)		

### 2.5 Inclusion criteria

Animal drug vendors were registered with either the VCN or the Kano State Veterinary Drugs and Agro-allied Association.

### 2.6 Exclusion criteria

- Non-Hausa-speaking vendors
- Participants below 18 years old
- Participants who were not available in business or traveled
- Participants who did not consent to the interview

### 2.7 Variables

In this study, AMR knowledge was defined as the vendors' understanding of antimicrobial resistance, including its causes, consequences, and prevention strategies (18). Practice was defined as the actual behaviors related to antimicrobial dispensing, including adherence to guidelines, handling of expired drugs, use of diagnostics, and participation in stewardship activities (19). These definitions guided how the constructs were operationalized and measured.

The primary outcome variables were the levels of AMR knowledge and AMR-related practices among animal drug vendors. The exposure variables included educational status, years in business, job role, AMR training, sources of AMR information, age, and gender. The potential confounders considered in the analysis were age, educational status, duration in business, and previous AMR training.

No diagnostic criteria were applied because the study did not involve clinical diagnosis.

Conceptual framework: the questionnaire and interpretation of vendors' dispensing behavior were informed by behavioral science frameworks, the Health Belief Model (HBM), the Theory of Planned Behavior (TPB), and the COM-B model, to capture perceived risk and benefits, social norms and intention, and capability–opportunity–motivation constraints that may shape antimicrobial dispensing practices.

### 2.8 Data collection and measurement

Data were gathered through a pretested questionnaire administered by interviewers, which was developed using KoboCollect version 2022.4.4 to ensure secure data entry and management. The questionnaire included 31 fair and unbiased questions, both close- and open-ended, organized into seven sections (A–G). It was constructed to gather information on sociodemographic characteristics, knowledge of antimicrobial resistance (AMR), practices related to antimicrobial use in animals, perceptions and attitudes toward AMR, factors influencing AMR-related practices, areas for improvement, and participant feedback.

The questionnaire was pretested on 20 animal drug vendors in Kebbi State, a location outside the primary study area, to avoid contamination of the main sample. The pretest participants were selected from the same target population as those in Kano (registered animal drug vendors) to ensure similarity. The pretest evaluated the clarity, language, structure, and flow of the questions. Based on the feedback, minor adjustments were made to improve wording and layout. The tool demonstrated satisfactory reliability, achieving a Cronbach's alpha of 0.728. To ensure consistency and accuracy, two independent transcribers cross-verified the translated responses.

The full study questionnaire is provided as Supplementary material S1. In addition, we provide an item-level summary table reporting the frequency of correct/incorrect responses for each knowledge item and recommended/non-recommended responses for each practice item to enable identification of specific gaps.

Data on demographics gathered included participants' age, gender, level of education, and the number of years they had worked as animal drug vendors.

To evaluate knowledge of AMR, questions explored causes, consequences, and modes of transmission. Correct answers were summed to generate a composite knowledge score. For inferential analysis, scores were dichotomised using a ≥70% threshold to indicate “adequate” knowledge. This cut-off has been widely applied in KAP studies as a pragmatic marker of minimum competency and supports comparability across studies, and it is consistent with instrument benchmarking approaches used in prior research and during our pilot testing (7).

Attitudes toward AMR prevention and control were assessed using Likert-scale items; responses were summed to form a composite attitude score and dichotomised at ≥70% to indicate a “positive” attitude and < 70% a “negative” attitude (7). Practices were evaluated through items on guideline adherence, management of expired products, client counseling, and use of diagnostics. Positive responses were aggregated into a composite practice score and classified using the same ≥70% benchmark to indicate “good” practice and < 70% “poor” practice (7).

To minimize bias, interviewers followed standardized procedures and avoided leading questions using a consistent interview guide. The questionnaire was translated into Hausa and backtranslated to English, with translations verified by two independent transcribers. Participants were assured of confidentiality, and interviews were conducted privately to reduce social desirability bias. All eligible vendors from the official registry were approached, with follow-ups for those initially unavailable. While the study relied on self-reported data, creating potential recall bias, questions were worded clearly and focused on recent behaviors to address this limitation.

### 2.9 Statistical methods

All data were analyzed using R statistical software. Descriptive statistics, including frequencies, percentages, means, and standard deviations, were used to summarize respondents' sociodemographic characteristics and knowledge and practices related to AMR.

For the bivariate analysis, chi-square tests were used to examine the associations between categorical variables, such as knowledge and practice levels, and demographic and exposure variables.

Statistical significance was set at *p* < 0.05.

Multivariable analysis was conducted using binary logistic regression to identify independent predictors of adequate AMR knowledge and practice. Only variables that were significant at *p* < 0.05 in the bivariate analysis and those considered theoretically relevant were included in the regression model. Adjusted odds ratios (AORs) with 95% confidence intervals (CIs) were reported to determine the strength of associations while controlling for potential confounders such as age, education level, and prior AMR training.

We additionally assessed an interaction term between years of experience and AMR-related training to examine whether the association between training and outcomes differed by experience level.

### 2.10 Ethical considerations

Ethical approval for this study was obtained from the National Health Research Ethics Committee of Nigeria (NHREC Approval Number: NHREC/17/03/2018) and the State Health Research Ethics Committee (Approval Number: SHREC/2024/5495). All methods were carried out in accordance with relevant guidelines and regulations. Informed consent was obtained from all participants prior to participation in the study, and confidentiality of all data was strictly maintained.

## 3 Results

### 3.1 Participants' socio-demographic characteristics

**Figure 1:**
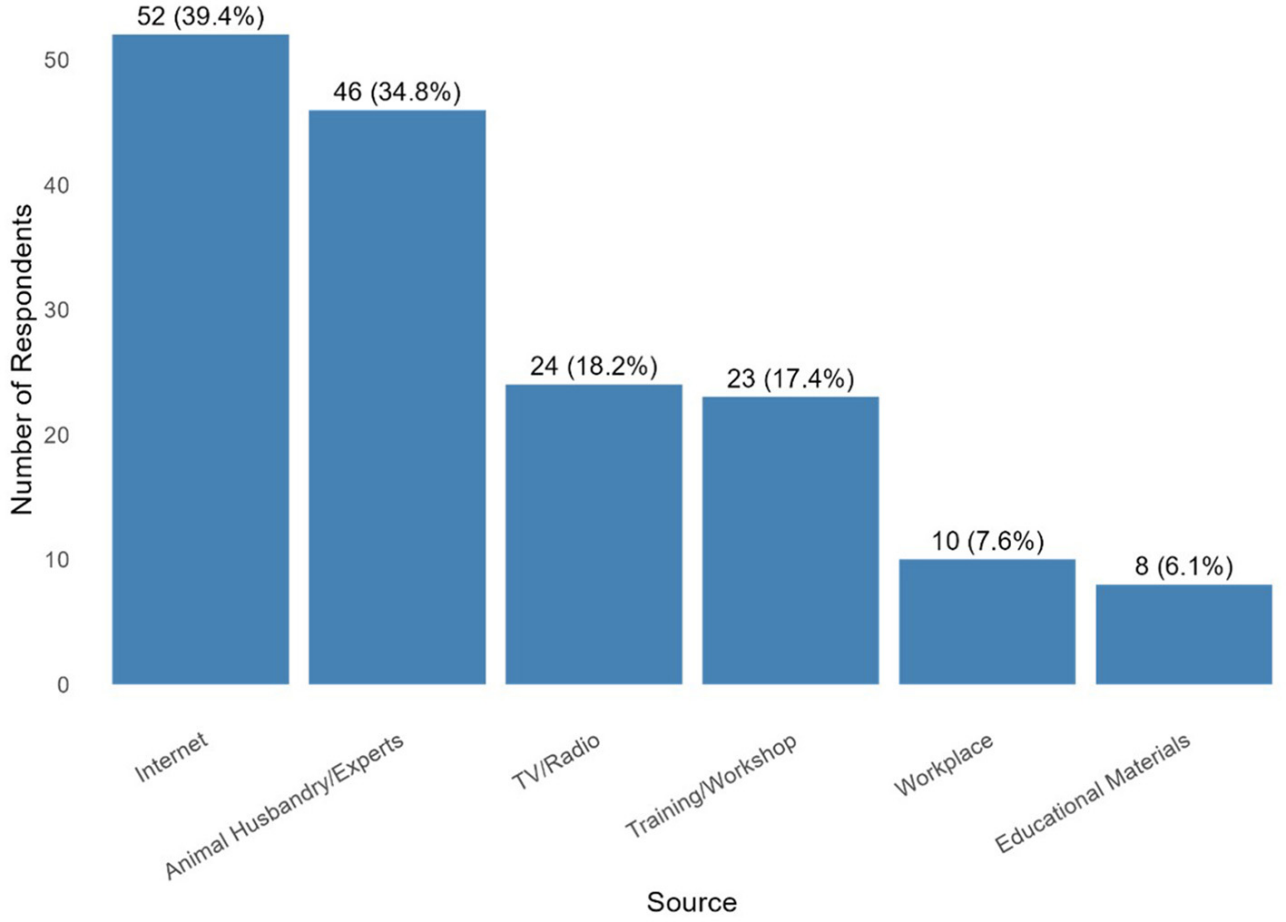
Sources of antimicrobial resistance information among animal drug vendors, Kano State.

The study population predominantly consisted of individuals aged 30–39 years (51.1%), with a mean age of 34.8 years (± 2.06). The majority were male (99.2%) and resided in urban areas (99.2%). Most participants sold animal drugs (98.5%) and held managerial roles (71.2%). The distribution of years in business was evenly split between 4 and 6 years and over 10 years (42.3% each) (Table 1). Sources of AMR information was mostly accessed through the internet (52%), while least accessed through educational materials (6.1%) (Figure 1).

The survey of 132 animal drug vendors revealed that 34.8% had adequate knowledge of antimicrobial resistance (AMR), while 65.2% had inadequate knowledge. In terms of attitude, 65.2% demonstrated a positive attitude toward AMR prevention and control, whereas 34.8% demonstrated a negative attitude. Regarding practices, only 23.5% reported good dispensing practices, while 76.5% demonstrated poor practices. Scores ≥70% were classified as adequate/positive/good as appropriate; scores < 70% were classified as inadequate/negative/poor (Table 2).

**Table 2:** Knowledge, attitude, and practice scores.

Variable (*n* = 132)	Frequency (%)
Knowledge level	
Good	46 (34.8)
Poor	86 (65.2)
Attitude level	
Good	86 (65.2)
Poor	46 (34.8)
Practice level	
Good	31 (23.5)
Poor	101 (76.5)

### 3.2 Association between sociodemographic characteristics and knowledge, attitude, and practices

The analysis in Table 3 revealed that education level was significantly associated with knowledge of antimicrobial resistance (AMR), with tertiary education linked to higher knowledge levels (*p* = 0.0155, χ^2^ = 5.36, df = 1, *p* = 0.0207). However, no significant associations were found between training status and practice level (χ^2^ = 2.30, df = 1, *p* = 0.1293) or between years of experience and knowledge level (χ^2^ = 1.45, df = 3, *p* = 0.6937).

**Table 3:** Associations between sociodemographic characteristics and KAP toward AMR.

Association tested	Test	*X* ^2^	df	*p*-value	Significance
Training status vs. practice level	Chi-square	2.3	1	0.1293	Not significant
Education level vs. knowledge level	Fisher's exact			0.0155	Significant
Tertiary vs. up to secondary (knowledge level)	Chi-square	5.36	1	0.0207	Significant
Years of experience vs. knowledge level	Chi-square	1.45	3	0.6937	Not significant

### 3.3 Multivariable analysis of factors associated with knowledge

In the multivariable logistic regression analysis (Table 4), vendors with up to secondary education had lower odds of good AMR knowledge compared with those with tertiary education (OR = 0.43, 95% CI: 0.20–0.94, *p* = 0.0347). Prior AMR-related training was associated with higher odds of good knowledge (OR = 2.21, 95% CI: 1.02–4.82, *p* = 0.0445). Years of experience was not significantly associated with knowledge (OR = 1.44, 95% CI: 0.67–3.18, *p* = 0.3601).

**Table 4:** Multivariable logistic regression analysis of factors associated with good knowledge of antimicrobial resistance among animal drug vendors.

Variable	OR	95% CI	*p*-value
Education level (tertiary vs. up to secondary)	0.43	0.20–0.94	0.0347
Training status (Yes vs. No)	2.21	1.02–4.82	0.0445
Years of experience ( ≤ 6 years vs. >6 years)	1.44	0.67–3.18	0.3601

The analysis indicates that vendors with tertiary education and prior training had better knowledge of antimicrobial resistance (AMR), emphasizing the role of formal education and targeted training in enhancing understanding. These findings suggest that improving educational opportunities and providing regular AMR-specific training could strengthen knowledge among animal drug vendors, promoting cautious antimicrobial use.

## 4 Discussion

This study identified important gaps in antimicrobial resistance (AMR) knowledge and practices among animal drug vendors in Kano State, Nigeria. While more than half of respondents demonstrated a positive attitude toward AMR prevention and control, only one-third had good knowledge, and fewer than one-quarter reported good practices. This misalignment between awareness and behavior highlights a critical weakness in antimicrobial stewardship at the community retail level.

For example, a study among agrovet staff in western Kenya found that while many respondents were aware of AMR risks, inappropriate dispensing practices remained common; clinical training was strongly associated with higher knowledge levels, a finding that parallels our observation that tertiary education and prior AMR-specific training significantly improved knowledge (20).

A multi-country assessment of KAP among livestock keepers and animal health providers in five African nations similarly reported substantial variability in AMR knowledge and practices. Vendors frequently operated without formal veterinary oversight and often sourced antimicrobials directly from suppliers, by-passing regulatory controls. The authors concluded that knowledge improvements alone may not be sufficient to change entrenched behaviors (21), an interpretation supported by our finding of persistently poor practices despite good attitudes. These studies suggest that knowledge improvement alone may be insufficient to change entrenched dispensing behaviors.

Studies on community animal health workers and paraprofessionals have shown persistent gaps in antimicrobial stewardship, with correct dosing occurring in fewer than half of dispensing events and frequent instances of non-prudent prescribing (22). These findings align with our results and reinforce the importance of targeted, tiered training for vendors. The observed association between prior training and improved knowledge in this study suggests that such interventions could be effective if delivered systematically and reinforced over time.

Regulatory and market factors also play a crucial role. Research in Uganda has highlighted how easy access to critically important antimicrobials and weak enforcement of prescription requirements contribute to inappropriate use (23). These structural constraints may partly explain why positive attitudes toward AMR among vendors in our study did not translate into better practices. Without complementary policy measures, such as enforcing prescription requirements, strengthening vendor licensing systems, and conducting routine inspections, training interventions alone are unlikely to produce sustained behavior change.

The influence of animal drug vendors extends beyond the point of sale to on-farm antimicrobial use. In poultry and mixed-livestock systems in Kenya, farmers frequently purchase antibiotics without prescriptions, self-administer treatments, and fail to observe withdrawal periods, often relying heavily on vendor guidance (24). Similar behaviors have been reported in Ghana, where agro-veterinary suppliers are key points of contact for livestock owners but operate with minimal regulatory oversight (25). These parallels highlight the pivotal role vendors play as intermediaries between regulatory systems and livestock producers, with direct implications for antimicrobial use patterns in the wider community.

Study limitations and generalisability: The non-response rate was high (43.6%), and participation depended on vendors' availability and willingness to be interviewed. This raises the possibility of non-response bias if vendors who declined differed systematically from those who participated (for example, in their dispensing behaviors or prior training exposure). Although all eligible vendors on the official registry were approached, the analytic sample may over-represent vendors who were more engaged with formal systems or more comfortable discussing their practices, which could lead to either underestimation or overestimation of true KAP levels.

In addition, recruitment was centered on a modern veterinary drug market in Kano metropolitan area and the sample was predominantly urban. Animal drug vending in rural or peri-urban settings may operate under different constraints, such as reduced access to veterinary oversight, diagnostics, and continuing education, potentially affecting stewardship behaviors. Accordingly, the findings should be generalized primarily to registered vendors operating in similar urban markets in Kano State. Future studies should use probability-based sampling across multiple markets and include rural dispensing points to improve representativeness and to quantify potential differences by setting.

Taken together, these findings point to the need for a multi-pronged public health approach to reducing AMR risks at the retail level. AMR-focused training should be embedded within state licensing requirements for animal drug vendors and reinforced through regular refresher courses. Implementation should be led by the Kano State Ministry of Agriculture and Natural Resources (Department of Veterinary Services), in collaboration with the Veterinary Council of Nigeria (VCN) and relevant vendor associations, with NCDC and other One Health partners providing technical support.

Embedding AMR competencies within initial licensing and periodic renewal offers a practical regulatory entry point for stewardship. These efforts should be complemented by stronger enforcement measures, including prescription-only sales, routine audits of dispensing practices, and supportive supervision by veterinary authorities. Beyond training alone, behaviorally informed strategies such as peer-to-peer monitoring within vendor associations, audit-and-feedback using simple dispensing scorecards, and mobile-based decision-support tools such as dosing guidance, referral prompts, and withdrawal-period reminders may help narrow the attitude–practice gap. Finally, linking vendor dispensing data with farm-level antimicrobial use would strengthen monitoring and enable assessment of whether these interventions translate into measurable improvements in practice.

In conclusion, AMR remains a major public health threat with disproportionate impacts in low- and middle-income countries. Our findings show that animal drug vendors in Kano State represent a critical point for strengthening antimicrobial stewardship at the animal–human interface. Embedding AMR competencies within licensing and renewal, alongside supportive regulation and practical decision support, offers a feasible pathway to improve dispensing practices and reduce inappropriate antimicrobial use.

## Data Availability

The raw data supporting the conclusions of this article will be made available by the authors, without undue reservation.
