# Supplementary material for: Antimicrobial resistance knowledge and practices among animal drug vendors in Kano State, Nigeria
Source: Front Public Health. 2026 Apr 10;14:1793310. doi: 10.3389/fpubh.2026.1793310 (PMC13106365; doi:10.3389/fpubh.2026.1793310)

Knowledge and Practices of Antimicrobial Resistance (AMR) Among Animal Drug Vendors in Kano state, 2024

Before you begin, we would like to inform you that your participation in this survey is voluntary. Your responses will be confidential and used only for research purposes. You have the right to withdraw at any time. By continuing, you are giving your consent to participate in this study

- ◯ Yes
- ◯ No

Section A: Sociodemographic Characteristics

I agree to provide the name of my registered business

- ◯ Yes
- ◯ No

If yes, please provide registered business name

____

1. Age as at last birthday

yyyy-mm-dd

____

2. Gender

◯ Male ◯ Female

3. What is your highest level of education?

- ◯ No formal education
- ◯ Completed primary school
- ◯ Completed secondary school
- ◯ Completed tertiary education (e.g., college, university)

4. How many years have you been selling animal drugs?

- ◯ Less than 1 year
- ◯ 1-3 years
- ◯ 4-6 years
- ◯ 7-10 years
- ◯ Over 10 years

5. Where do you live presently?

◯ Urban area ◯ Rural area

6. What is your role in the business?

- [ ] Owner
- [ ] Employee
- [ ] Manager
- [ ] Others

6b. Others, please specify

---

7. Do you sell only animal drugs, or do you also sell human drugs?

- ◯ Only animal drugs
- ◯ Both animal and human drugs

Section B: Knowledge of Antimicrobial Resistance (AMR)

8. How would you define AMR?

---

9. Have you heard of Antimicrobial Resistance (AMR)?

- ◯ Yes
- ◯ No

9b. If yes, where did you hear about AMR?
(Select all that apply)

- [ ] Training/Workshop
- [ ] Veterinary professionals
- [ ] Other vendors
- [ ] Media (TV, Radio, Newspapers)
- [ ] Internet/Social Media
- [ ] Government/NGO campaigns
- [ ] Educational materials (pamphlets, brochures)

10. Do you know what happens when AMR occurs?

- ◯ Yes
- ◯ No

If yes, can you explain what you think are the consequences of antimicrobial resistance for animals, humans, and the community?

---

11. Do you know the consequences of AMR?

- ◯ Yes
- ◯ No

11b. What are the consequences of AMR?
(Select all that apply)

- ☐ Antibiotics may not work to treat infections
- ☐ Resistant bacteria may spread more
- ☐ Animal treatment may become more expensive
- ☐ More animals may die
- ☐ Resistant bacteria may spread to humans
- ☐ It can spread to the environment

12. Do you know of any rules for using antibiotics in animals?

- ◯ Yes
- ◯ No

12b. If yes, name them

____

13. How often do you sell antibiotics?

- ◯ Daily
- ◯ Weekly
- ◯ Monthly
- ◯ Rarely

Section C: Practices Regarding Antibiotic Use in animals

14. Do you follow any rules when giving antibiotics to animals?

- ◯ Yes
- ◯ No

14b. If yes, name them

____

15. Do you often provide guidance to customers on the proper use of antibiotics including dosage, duration and adherence?

- ◯ Never
- ◯ Rarely
- ◯ Sometimes
- ◯ Often
- ◯ Always

16. Do you check the expiry date of drugs before selling?

- ◯ Always
- ◯ Sometimes
- ◯ Never
- ◯ Rarely
- ◯ Often

17. What do you do with expired or low-quality antibiotics?

- ◯ Burn them
- ◯ Return to supplier
- ◯ Throw in a waste bin
- ◯ Use them anyway
- ◯ Others

17b. Others(Please specify)

---

18. What do you do if a customer wants to buy antibiotics without a prescription?

- ◯ I sell the antibiotics without asking questions
- ◯ I ask the customer questions about their condition before deciding the antibiotics to give
- ◯ I refuse to sell and explain the importance of a prescription
- ◯ I suggest they see a veterinarian first
- ◯ Please name them;

18b. Others, please specify

---

19. Do you give recommendations/advice to customers when selling antibiotics?

- ◯ Yes
- ◯ No

19b. What recommendations do you give to customers when selling antibiotics?
(Select all that apply)

- ☐ Correct dosage and duration of treatment
- ☐ Complete the full course of antibiotics
- ☐ Possible side effects of the antibiotics
- ☐ Warning against misuse (e.g., using for other conditions)
- ☐ Storage instructions for the drugs
- ☐ Others;

19c. Others, please specify

---

20. Have you received any training on the proper use of antibiotics?

- ◯ Yes
- ◯ No

20b. If yes, specify...

Section D: Perception and Attitudes Towards AMR

21. How serious is AMR in your community?

- ◯ Not serious at all
- ◯ Slightly serious
- ◯ Moderately serious
- ◯ Serious
- ◯ Very serious

22. Do you think vendors like you can help control AMR?

- ◯ Strongly disagree
- ◯ Disagree
- ◯ Neutral
- ◯ Agree
- ◯ Strongly agree

If you agree, in what ways do you think you can contribute to controlling AMR?

____

If you disagree, what are the barriers that prevent you from contributing?

____

23. How confident are you in advising customers on the proper use of antibiotics?

- ◯ Not confident at all
- ◯ Slightly confident
- ◯ Neutral
- ◯ Confident
- ◯ Very confident

24. What challenges do you face in selling antibiotics responsibly?
(Select all that apply)

- ☐ High customer demand for antibiotics
- ☐ Lack of awareness or knowledge about AMR
- ☐ Competition with other vendors
- ☐ Economic pressures (e.g., need to make sales)
- ☐ Lack of veterinary support
- ☐ Limited access to reliable information
- ☐ Weak regulations or enforcement
- ☐ If others;

Please specify

____

25. What can help reduce AMR in the veterinary sector?
(Select all that apply)

- ☐ Better training for vendors
- ☐ Stricter enforcement of regulations
- ☐ Increased public awareness
- ☐ Better access to veterinary services
- ☐ Improved quality control of drugs
- ☐ Regular monitoring and evaluation
- ☐ Others

25b. Others, please specify

____

Section E: Factors Influencing AMR-Related Practices

26. What factors influence your decision to recommend specific antibiotics?
(Select all that apply)

- ☐ Cost of the drug
- ☐ Availability of the drug
- ☐ Customer preference
- ☐ Training received
- ☐ Influence of suppliers
- ☐ Recommendations from other colleagues
- ☐ Other factors;

26b. Others, please specify

____

27. Does the cost of antibiotics affect your ability to follow AMR guidelines?

- ◯ Yes
- ◯ No

28. Do you know of any regulatory bodies that monitor AMR practices?

- ◯ Yes
- ◯ No

28b. If yes, Please name of the regulatory body

____

Section F: Identification of Gaps and Areas for Improvement

29. What challenges do you face in following AMR guidelines? (Select all that apply)

- ☐ Lack of knowledge
- ☐ High cost of AMR-compliant drugs
- ☐ Lack of training opportunities
- ☐ Weak enforcement of regulations
- ☐ Other challenges

29b. Please specify

____

30. What support do you need to improve your AMR practices? (Select all that apply)

- ☐ Access to training programs
- ☐ Financial incentives
- ☐ Stronger regulations
- ☐ Better access to AMR-compliant drugs
- ☐ Public awareness campaigns
- ☐ Other support;

30b. Others, please specify

____

Section G: Feedback and Suggestions

31. Do you have any suggestions for improving AMR control among animal drug vendors in Nigeria?

○ Yes ○ No

Yes, please specify

____

Please take GPS Point

p

latitude (x.y °)

____

longitude (x.y °)

____

altitude (m)

____

accuracy (m)

____

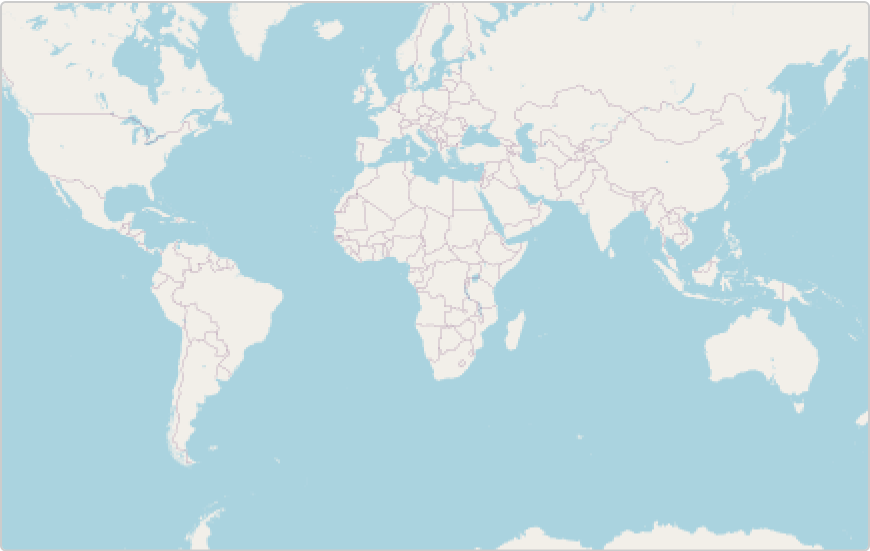

Supplement: Supplementary file 1 [file Data_Sheet_1.pdf]
